# MALAT1 accelerates the development and progression of renal cell carcinoma by decreasing the expression of miR‐203 and promoting the expression of *BIRC5*

**DOI:** 10.1111/cpr.12640

**Published:** 2019-06-27

**Authors:** Haimin Zhang, Wei Li, Wenyu Gu, Yang Yan, Xudong Yao, Junhua Zheng

**Affiliations:** ^1^ Department of Urology, Shanghai Tenth People’s Hospital Tongji University School of Medicine Shanghai China; ^2^ Department of Urology, Shanghai General Hospital The First People's Hospital Affiliated to Shanghai Jiaotong University Shanghai China

**Keywords:** *BIRC5*, MALAT1, miR‐203, renal cell carcinoma

## Abstract

**Objective:**

We aimed to investigate the roles of the lncRNA MALAT1 in renal cell carcinoma (RCC) progression.

**Methods:**

qRT‐PCR was used for the assessment of *BIRC5*, miRNA‐203 and MALAT1 expression. Furthermore, the targeted relationships between miR‐203 and *BIRC5*, as well as MALAT1 and miR‐203, were predicted by the miRanda/starBase database and verified by dual‐luciferase reporter gene assay. The effects of MALAT1, miRNA‐203 and *BIRC5* on cell proliferation, cell cycle, cell apoptosis, cell invasion and cell migration were studied by using CCK‐8, flow cytometry, transwell and wound healing assays, respectively. In addition, the effects of MALAT1 on RCC tumorigenesis were evaluated in vivo by nude mouse tumorigenesis.

**Results:**

The expression levels of *BIRC5* and MALAT1 were higher in RCC tissues and cell lines than in adjacent normal tissues and a normal renal cortex proximal tubule epithelial cell line. In contrast, the expression of miRNA‐203 in RCC tissues and cell lines was higher than that in adjacent normal tissues and a normal renal cortex proximal tubule epithelial cell line. *BIRC5* and MALAT1 promoted cell proliferation yet decreased the percentage of RCC cells at G0/G1 phase.

**Conclusions:**

Our study demonstrated that MALAT1 functions as a miR‐203 decoy to increase *BIRC5* expression in RCC.

## 1 INTRODUCTION

Renal cell carcinoma (RCC) is a common cancer that accounts for 2%‐3% of all cancerous diseases in adults.^1^ There are approximately 65 000 cases of RCC each year, and RCC is the eighth most common cause of cancer mortality.^2^ RCC affects quality of life and life expectancy and has important health and economic implications related to metabolic syndromes, increased cardiovascular risk and end‐stage kidney disease.^3^ In addition, clear cell renal cell carcinoma (ccRCC) is the most universal subtype of RCC, accounting for approximately 75% of RCC. Furthermore, the morbidity and mortality rates of RCC are rising globally.^4^ Besides traditional surgery, RCC is resistant to the other forms of therapies chemotherapy and radiotherapy. The 5‐year survival rate of RCC is approximately 55%, while the 5‐year survival rate of metastatic RCC is approximately 10%.^5^ Patients with metastatic RCC are faced with a depressing prognosis and limited therapeutic options. The median survival time in a recent cohort study was only 1.5 years, and the survival rate was less than 10% in patients who survived 5 years.^6^ Thus, it is vital to study the molecular basis of RCC to design novel therapeutic drugs to improve survival rates.

MicroRNAs (miRNAs) are 19‐22 nucleotide‐long non‐coding RNAs that function as negative regulators of translation and are involved in many cellular processes. Increased levels of specific miRNAs have been closely related to a variety of diseases, such as cancers, diabetes, obesity and cardiovascular disease.^7^ miRNAs are non‐coding RNAs, and it has been estimated that 30% of all genes in animals are regulated by translational and post‐transcriptional repression, cleavage or destabilization.^8^ miRNA dysregulation is an important component of this landscape, which relies on both the oncogenic and tumour‐suppressive functions of miRNAs. Among miRNAs, the highly conserved let‐7 family has a prominent role in regulating embryonic development and the maintenance of differentiated tissues. Let‐7 is as a potent tumour suppressor via its post‐transcriptional repression of multiple oncogenes including RAS, Myc and HMGA2. The let‐7 family is downregulated in multiple tumour types and causally linked to oncogenesis.^9^ miRNAs are also aberrantly expressed in several eukaryotic organisms to regulate the stability and processing of target mRNA through directly binding to 3′UTRs. miRNAs have been reported to be involved in cell proliferation, cell differentiation and cell apoptosis, resulting in a reduction in the microRNA levels of hundreds of small target mRNAs.^10^ miR‐203 is a tumour suppressor in a variety of human cancers, such as hepatocellular carcinoma (HCC),^11^ prostate cancer^12^ and liver cancer.^13^ Besides, miR‐203 can directly suppress the expression of transcription factor p63 during epidermal differentiation, thus limiting the proliferation potential and inducing the withdrawal of the cell cycle to eventually promote epidermal differentiation.^14^ In rhabdomyosarcoma cells, the overexpression of miR‐203 suppressed cell growth and promoted myogenic differentiation.^15^

Currently, lncRNAs are differentially expressed in various tissues and have essential functions in gene regulatory processes in normal cells and cancer cells. Furthermore, many lncRNAs are associated with chromatin modification complexes and act as miRNA sponges, which adjust gene expression.^16^ LncRNAs are the largest class of non‐coding RNAs. Non‐coding RNAs, once thought to be part of the transcriptional noise, now constitute a regulatory layer of transcriptional and post‐transcriptional regulation. The enhanced transcriptional noise and gene expression regulatory function of lncRNAs are fully supported by their functional roles observed in various important biological environments.^17^ In previous reports, high expression levels and the acute hypoxic induction of MALAT1 in several mouse organs suggested a hitherto unrecognized role of this lncRNA in systemic adaptation hypoxia.^18^ In addition, the upregulation of MALAT1 was correlated with cancer progression and poor prognosis in clear cell renal cell carcinoma.^19^ However, the roles of MALAT1 in RCC progression need to be further understood.

*BIRC5* (also known as survivin) is a critical anti‐apoptotic protein that is been involved in many cancer types. *BIRC5* inhibits apoptosis‐related signalling pathways and promotes cell proliferation to affect cancer progression.^20^ *BIRC5*, which encodes surviving, is upregulated in both adenocarcinoma and squamous cell carcinoma tissues, and the high expression of *BIRC5* is related to poor survival in adenocarcinoma, but not squamous cell carcinoma. In addition, survivin was identified as a candidate marker of aggressiveness in clear cell renal cell carcinoma (ccRCC), and high expression levels of survivin protein predicted a poor outcome for ccRCC patients.^21^ In addition, the ratio of the miR‐195 level to the *BIRC5* level was associated with both recurrence‐free and overall survival in lung adenocarcinoma.^22^ Previous researches showed that the miR‐195/*BIRC5* axis is a potential target for the specific treatment of lung adenocarcinoma, especially for NSCLC (non‐small‐cell lung carcinoma).^22^ *BIRC5* is a new member of inhibitor of IAP family, the proteins of which regulate the cell cycle and apoptosis. Besides, the expression of *BIRC5* was induced by hypoxia,^23^ and *BIRC5* promoted angiogenesis and was strongly correlated with cell proliferation.^24^ There is increasing evidence that indicated that *BIRC5* is highly expressed in most human tumours and closely related to tumour progression, tumour recurrence, chemotherapy resistance and poor prognosis.^25^, ^26^

The aim of our study was to investigate the roles of MALAT1/miR‐203/*BIRC5* in the development and progression of RCC, which might provide us with more diagnostic and therapeutic strategies for RCC in the future.

## 2 MATERIALS AND METHODS

### 2.1 Clinical samples

Seventy human RCC tissue and adjacent normal kidney tissues samples were obtained from patients with a pathological and cytological diagnosis of RCC in Shanghai General Hospital, The First People's Hospital Affiliated to Shanghai Jiaotong University. Adjacent normal tissues 2 cm away from the RCC tissues were selected and excised to be used as our experimental materials. Tumorous and normal regions were confirmed by three pathologists before the experiments. The renal tumour specimen type was confirmed based on immunohistochemistry (IHC), histological evaluation and TNM (tumour‐node‐metastasis) staging. Clinical information is shown in Table 1. The expression level of *BIRC5* was defined based on the results of qRT‐PCR. The expression level of *BIRC5* in normal tissues was set as the threshold. The tumour and paired normal kidney samples were immediately frozen in liquid nitrogen. Patients in this study signed informed consent forms and agreed that their samples could be used for experimental studies. Our protocol was approved by the Ethics Committee of Shanghai General Hospital, The First People's Hospital Affiliated to Shanghai Jiaotong University.

**Table 1:** Characteristics of patients (N = 70)

Variable	RCC	
	No.	%
Sex		
Male	43	61.43
Female	27	38.57
Age at diagnosis, y		
Median	64	
Range	51‐75	
Tumour size (cm)		
<7	23	32.86
≥7	47	67.14
TNM stage		
I and II	26	37.14
III and IV	44	62.86
Fuhrman grade		
Grades 1 and 2	19	27.14
Grades 3 and 4	51	72.86
Lymph node metastasis		
Negative	25	35.71
Positive	45	64.29
BIRC5 expression		
High	63	90.00
Low	7	10.00

Abbreviation: RCC, renal cell carcinoma.

### 2.2 Cell culture

The normal proximal tubule epithelial cell line HK‐2, RCC cells lines (A498, 786‐O, OS‐RC‐2 and CAKI‐1) and the HEK293T cell line were all bought from BeNa Culture Collection. HK‐2, HEK293T and 786‐O cells were kept in DMEM‐H (Shenzhen Hongyi Long Import and Export Co., Ltd). MEM‐EBSS was used to cultivate A498 cells. OS‐RC‐2 cells were cultured in RPMI1640 (BioSun), and the CAKI‐1 cell line was kept in ATCC‐formulated McCoy′s 5A medium (Thermo Fisher). All media were supplemented with 10% foetal bovine serum (FBS) and kept at 37°C in a 5% CO_2_ atmosphere.

### 2.3 Immunohistochemistry (IHC)

The tissue sections were dried at 60°C for 1 hour and then dewaxed by an automatic dyeing machine. The tissue sections were incubated after being washed with PBS with 3% hydrogen peroxide at room temperature for 6 minutes. The sections were then immersed in 0.01 M 3% citrate buffer. Afterwards, they were heated at 95°C for 10 minutes in a microwave and cooled to room temperature. After 30 minutes, non‐immune goat serum was added and the sections were incubated overnight with BIRC5 (1:1000 v/v) and Ki‐67 (1:300 v/v) (Abcam) at 4°C. Afterwards, they were washed in PBS, labelled with HRP‐labelled goat anti‐rabbit IgG (Abcam) (1:1000 v/v) and incubated for 30 minutes at room temperature. Besides, the sections should be exposed to freshly prepared diaminobenzidine and stained for 4‐6 minutes. The sections were also stained for 15 seconds with haematoxylin. Finally, the sections were rinsed with water.

### 2.4 Quantitative real‐time PCR (qRT‐PCR)

Total RNA in the RCC tissue samples and tumour cells were extracted with TRIzol reagent (Invitrogen) according to manufacturer's instructions. For each sample, the amount of the total RNA was determined to be 200 ng by a NanoDrop 2000 (Thermo Fisher Scientific). A reverTra Ace qPCR RT Kit was employed for reverse transcription of RNA. Three groups of lncRNAs were calibrated by qRT‐PCR using the THUNDERBIRD SYBR® qPCR Mix (Toyobo). The reaction conditions and steps were as follows: 94°C for 2 minutes, 94°C for 10 seconds, 56°C for 30 seconds, 72°C for 1 minute and 72°C for 10 minutes. GAPDH and U6 were used as internal loading controls. qRT‐PCR was repeated at least three times. The expression levels of the mRNAs and lncRNAs were normalized against those of GAPDH and relatively quantified using the $2^{-\Delta \Delta {\text{C}}_{\text{t}}}$ method. The expression of the miRNAs was normalized against that of U6 and relatively quantified using the $2^{-\Delta \Delta {\text{C}}_{\text{t}}}$ method. All the primers used for qRT‐PCR in this study are listed in Table S1.

### 2.5 Cell transfection and cultivation

siRNAs for MALAT1 or *BIRC5*, scrambled siRNAs, miR‐203 mimics, miR‐203 NC mimics, miR‐203 inhibitor and miR‐203 NC inhibitor were generated by GenePharma. The pcDNA3.1 plasmid purchased from Thermo Fisher Scientific and employed to overexpress MALAT1 or *BIRC5*. Before transfection, A498 cells and OS‐RC‐2 cells were digested with 0.25% trypsin and seeded in 6‐well plates (1 × 10^5 ^cells/well). When the cells reached 80%‐90% confluence, the initial medium was replaced with fresh serum‐free medium and antibiotics. Lipofectamine 2000 (Life Technologies Corporation, Gaithersburg, MD, USA) was used for transfection, and the transfected cells were cultivated at 37°C in a 5% CO_2 _atmosphere. The transfection efficiency was detected 48 hours after transfection. The sequences of the siRNA, mimics and inhibitor are given in Table S2.

### 2.6 Survival analysis

Survival probabilities were estimated by the Kaplan‐Meier plot method according to the website oncLnc (://www.oncolnc.org/). We calculated probabilistic survival estimates using probability‐stratified multiplicative statutory laws. The overall survival rate (OS) was predicted through fitting a univariate Cox regression model based on the anatomical stage and Fuhrman scale.

### 2.7 Dual‐luciferase reporter gene assay

The targeted relationship between miR‐203 and *BIRC5* was predicted by the miRanda database (http://34.236.212.39/microrna/). There were two potential binding sites between MALAT1 and miR‐203 according to starBase (://starbase.sysu.edu.cn/). The primers used in this study for amplification of *BIRC5* were as follows: F: TCTAGAGGCTGAAGTCTGGCGTAAGATGAT, R: TCTAGATAGATGAGTACAGAGGCTGGAGTGC.

The primers used in this study for the amplification of MALAT1 were as follows: F: TCTAGAAGAGGCAATGTCCATCTCAAAATAC, R: TCTAGATGATAAACTCACTGCAAGGTCTC. XbaI was employed for enzyme digestion in the amplification of the 3′UTRs of *BIRC5* and MALAT1. The pGL3‐control luciferase reporter gene vector (Promega, Madison, WI, USA) loaded with either MALAT1‐wt or MALAT1‐mut was co‐transfected with miR‐203 mimics or control into HEK293T cells using Lipofectamine 2000 reagent (Invitrogen). Similarly, the pGL3 luciferase reporter gene vector (Promega) loaded with either *BIRC5*‐wt or *BIRC5*‐mut was co‐transfected with miR‐203 mimics or control into HEK293T cells using Lipofectamine 2000 reagent (Invitrogen). The luciferase activities in cell lysates were measured with a Dual‐Glo Luciferase Assay System (Promega) 48 hours after transfection (Promega) in accordance with the manufacturer's instructions. Each experiment was repeated at least three times.

### 2.8 Cell proliferation (CCK‐8) assay

After the cells were transfected for 0, 24, 48 and 72 hours, CCK‐8 solution was added (10 μL, Dojindo) to each well. Thereafter, the RCC cells were incubated for 2 hours at 37°C in a 5% CO_2_ humidified chamber. Afterwards, the OD at 450 nm (OD450) was measured with a plate luminometer (Bio‐Rad). The experiment was repeated at least three times.

### 2.9 Flow cytometry analysis

For cell cycle experiments, different groups of cells were collected 72 hours after transfection and digested to obtain a cell suspension. Cell suspension was centrifuged, and the supernatant was discarded. Cells were washed twice with PBS to remove any residue and fixed in 75% ethanol at 4°C for 4 hours. Besides, the fixed cells were centrifuged and washed with PBS three times. Afterwards, 40 μg propidium iodide (PI) and 1 mL of a 100 μg RNase staining solution (BD Biosciences) were added to the fixed cells and the fixed cells were incubated for 15 minutes at room temperature in a dark area. A FACSCalibur flow cytometer was employed to detect the cell cycle after staining, and FACSDiva was utilized to analyse the statistical data. For apoptosis experiments, transfected cells from each group were harvested and digested with 0.25% trypsin after 72 hours. Afterwards, they were seeded into 96‐well culture plates at a density of 20 000 cells/well. 200 μL of HEPES, 5 μL of Annexin V/FITC and 5 μL PI were added to each well, and the reaction proceeded for 15 minutes at room temperature. Apoptosis was observed using a FACSCalibur FCM (BD Biosciences). Three independent experiments were conducted to reduce errors. Data analysis was conducted by FACSDiva software.

### 2.10 Wound healing assay

Cell motility was evaluated using a wound healing assay. RCC cells were plated in 24‐well plates with serum‐free medium until they reached 80% to 90% confluence. The cell monolayers were scratched across the centre of each well with a 10 μL micropipette tip. Wound healing was monitored at the indicated time points by phase‐contrast microscopy with a 20 × objective and an inverted microscope. The experiment was performed at least three times.

### 2.11 Transwell assay

Matrigel (BD Bioscience) and serum‐free DMEM were thoroughly mixed and placed in a transwell chamber (Corning Incorporated). Cells at a concentration of 2.5 × 10^4 ^cells/mL in serum‐free medium were placed into the upper chamber (500 μL for each chamber) of 24‐well invasion chambers, while culture medium containing 20% FBS was added to the lower chamber. A 4% paraformaldehyde solution was utilized to immobilize the cells, and 0.1% crystal violet was used to stain RCC cells after 24‐48 hours of incubation. Pictures were taken of each chamber, and the cells in 5‐10 independent fields were counted.

### 2.12 Western blot analysis

The concentration of each protein was determined using a Pierce BCA Protein Assay Kit (Pierce). After they were separated by 10% SDS‐polyacrylamide gel electrophoresis (SDS‐PAGE), the proteins were then transferred to polyvinylidene difluoride membrane (PVDF, Millipore, Billerica) for 120 minutes. Thereafter, membranes were blocked with TBST containing 5% skim milk and incubated with the primary antibody (anti‐*BIRC5*, ab76424, 1:5000; anti‐GAPDH, ab181602, 1:10 000). Subsequently, the membranes were incubated with a secondary antibody (anti‐rabbit IgG H&L, ab6721, 1:10 000) for 1 hour at 37°C. All of the antibodies in this study were purchased from Abcam. Signal detection was conducted by the ECL system (Life Technology). The relative protein levels among the samples using the GAPDH density as an internal loading control were compared.

### 2.13 Nude mouse tumorigenesis experiment

BALB/c nude mice were obtained from Shanghai General Hospital, the First People's Hospital Affiliated to Shanghai Jiaotong University. The mice were given free access to sterile food and water during the whole experimental process. All nude mice animal experiments strictly followed the rules of a programme approved by Shanghai General Hospital, the First People's Hospital Affiliated to Shanghai Jiaotong University. Female athymic BALB/c nude mice at their subcutaneous tissue of the right flank were injected with p‐MALAT1‐ or si‐MALAT1‐1‐transfected A498 cells (six nude mice per group), and six nude mice were injected with si‐control‐transfected A498 cells as a control. Tumour growth was monitored by two‐dimensional measurements using electronic callipers starting from the seventh day after tumour transplantation (once every 7 days for a total of 35 days).

### 2.14 Statistical analysis

Data are expressed as mean ± standard deviation (SD) from triplicate independent experiments. Student′s t test was used to compare differences in the two different groups with parametric variables. For three or more groups, difference analysis was performed by one‐way ANOVA. Statistical analysis was carried out with GraphPad Prism 6. *P* values <0.05 were considered as statistically significant.

## 3 RESULTS

### 3.1 *BIRC5* was overexpressed in RCC tissues and cells

**Figure 1:**
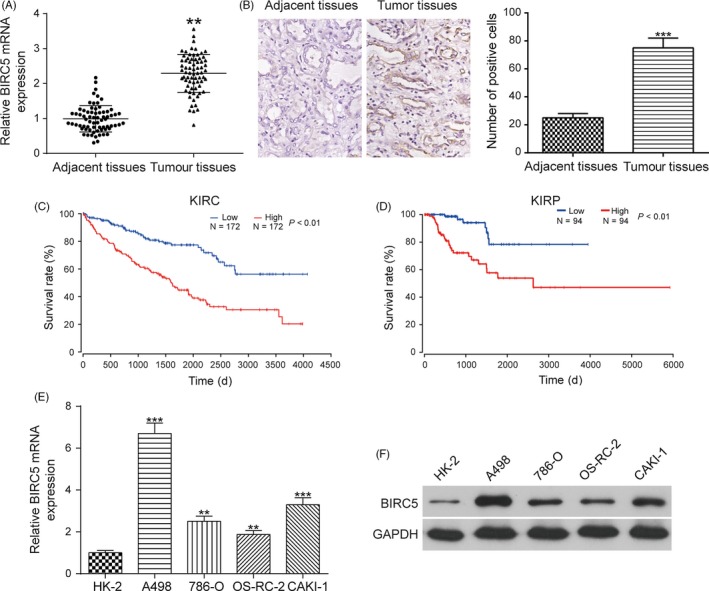
*BIRC5* expression was higher in RCC tissues and cell lines than in control tissues and cell lines. A, The expression of *BIRC5* was higher in RCC tissues than in adjacent normal tissues, as shown by qRT‐PCR. B, IHC results showed that the expression of *BIRC5* was higher in RCC tissues than in adjacent normal tissues. C and D, Kaplan‐Meier plot analysis illustrated that patients expressing high levels of *BIRC5* presented a significantly poorer prognosis than those expressing low levels of *BIRC5*. E and F, The increased expression levels of *BIRC5* were detected in four RCC cell lines (A498, 786‐O, OS‐RC‐2 and CAKI‐1) compared with its expression in a normal renal cortex proximal tubule epithelial cell line. Data were expressed as mean ± standard deviation of three independent experiments. **P < 0.01. ***P < 0.001

The mRNA and protein expression of *BIRC5* was higher in RCC tissues than in adjacent normal tissues as shown in Figure 1A,B. KIRC (kidney renal clear cell carcinoma) is the most common type of renal cell carcinoma, accounting for 70%‐80% of all renal cell carcinoma cases.^27^ KIRP (kidney renal papillary cell carcinoma) is the second most common histological subtype of RCC (renal cell carcinoma), and it accounts for 10% to 15% of all RCCs.^28^ The results of survival analysis illustrated that KIRC and KIRP patients expressing high levels of *BIRC5* presented a significantly poorer prognosis than those expressing low levels of *BIRC5* (Figure 1C,D). In addition, the expression level of *BIRC5* in four RCC cell lines (A498, 786‐O, OS‐RC‐2 and CAKI‐1) was higher than that in the normal renal cortex proximal tubule epithelial cell line HK‐2 (Figure 1E,F). In addition, the effects of *BIRC5* on cell function were explored. *P‐BIRC5* upregulated the expression of *BIRC5,* which was instead downregulated by si‐*BIRC5*‐1 and si‐*BIRC5*‐2 in both A498 and OS‐RC‐2 cell lines (Figure S1A‐B). Given that the knockdown efficiency was higher in the si‐*BIRC5*‐2 group than in the si‐*BIRC5*‐1 group, si‐*BIRC5*‐2 was utilized for further studies. The CCK‐8, flow cytometry, transwell and wound healing assays indicated that the overexpression of *BIRC5* greatly accelerated cell proliferation (Figure S1C‐D), decreased the percentage of RCC cells in G0/G1 phase (Figure S1E‐H, *P < 0.05) and promoted cell invasion (Figure S2A‐D, **P < 0.01, ##*P* < 0.01) and cell migration (Figure S2E‐H, **P < 0.01, ##*P* < 0.01) in both A498 and OS‐RC‐2 cells, respectively. The inhibition of *BIRC5* played an opposite role. Taken together, these data suggest that *BIRC5* was overexpressed in RCC tissues and cells, and *BIRC5* promoted the development and progression of RCC cells.

### 3.2 MiR‐203 directly targeted *BIRC5* and suppressed the expression of *BIRC5*

**Figure 2:**
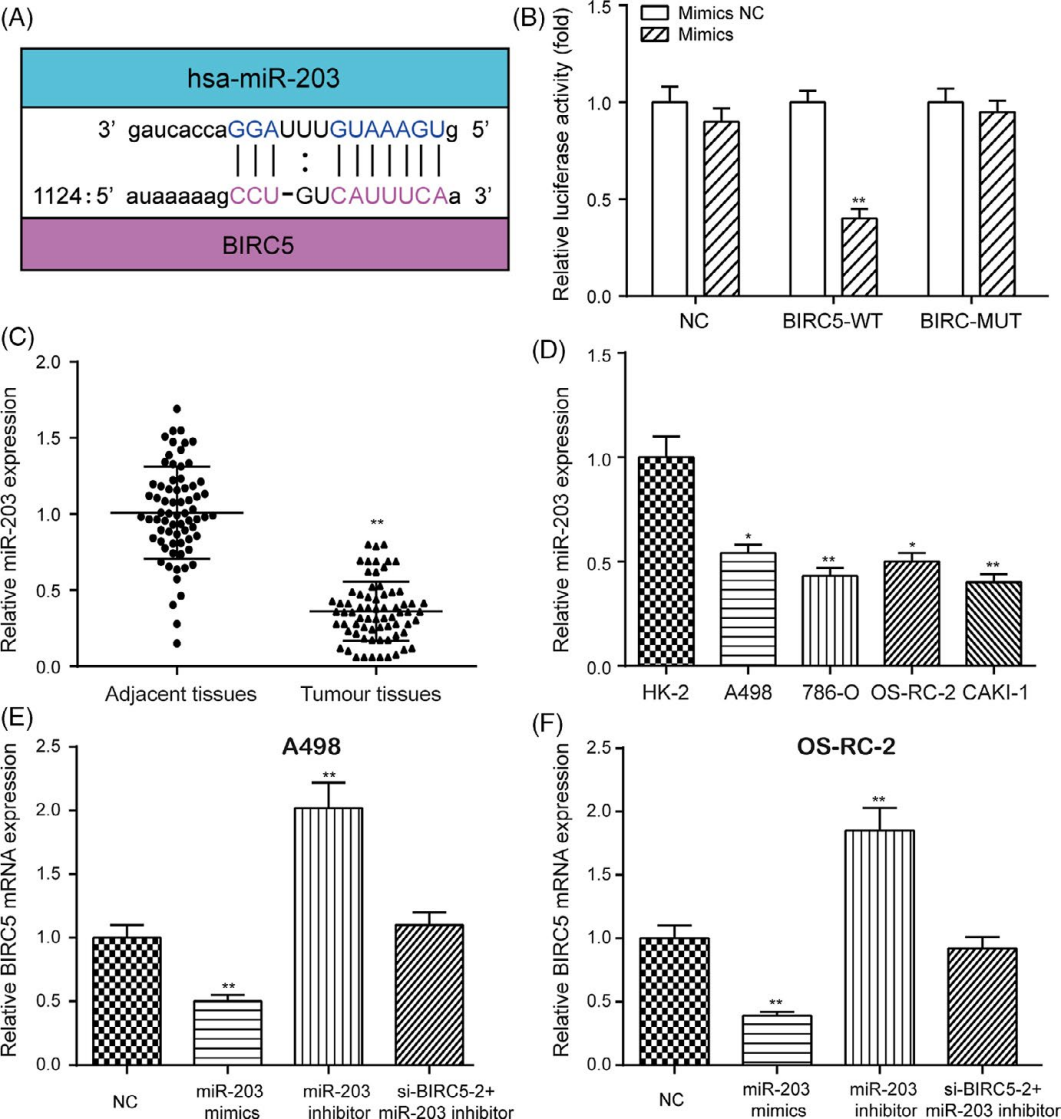
The targeted relationship between miR‐203 and *BIRC5* was validated. A, miR‐203 was predicted to target *BIRC5* by miRanda. B, Dual‐luciferase reporter gene assay validating the targeted relationship between miR‐203 and *BIRC5*. C, D, The expression of miR‐203 in RCC tissues and cell lines was examined by qRT‐PCR. E and F, Overexpression of miR‐203 decreased the expression of *BIRC5,* which was instead increased with downregulation of miR‐203 in the RCC cell lines A498 and OS‐RC‐2. Data were expressed as mean ± standard deviation of three independent experiments. *P < 0.05. **P < 0.01

The miRanda database predicted that that *BIRC5* is a potential target of miR‐203 (Figure 2A). To verify the targeted relationship, a dual‐luciferase reporter gene assay was employed. Dual‐luciferase reporter gene assay showed that the luciferase activity of the group co‐transfected with miR‐203 mimics and *BIRC5*‐wt was lower than that of the group co‐transfected with miR‐203 NC and *BIRC5*‐wt (Figure 2B, **P < 0.01), whereas co‐transfection with *BIRC5*‐mut did not affect luciferase activity, indicating that miR‐203 directly binds to *BIRC5* in RCC. miR‐203 expression was greatly downregulated in RCC tissues and cell lines compared with its expression in adjacent normal tissues and a normal renal cortex proximal tubule epithelial cell line (Figure 2C,D, *P < 0.05, **P < 0.01). Besides, transfection with miR‐203 mimics greatly upregulated the expression of miR‐203, which was downregulated with transfection with an miR‐203 inhibitor in both A498 and OS‐RC‐2 cells (Figure S3A, **P < 0.01). The qRT‐PCR results showed that the miR‐203 inhibitor upregulated the expression of *BIRC5,* which was instead downregulated by transfection with miR‐203 mimics in both A498 and OS‐RC‐2 cells (Figure 2E,F, **P < 0.01). The miR‐203 inhibitor increased the expression of *BIRC5*, which returned to its normal expression level after co‐transfected with si‐*BIRC5*‐2. Besides, regression analysis revealed that the expression of miR‐203 was negatively correlated with the expression of *BIRC5* in RCC (Figure S3B). In brief, miR‐203 directly targeted *BIRC5* and suppressed its expression in RCC.

### 3.3 Effects of miR‐203 on cell proliferation, cell cycle, apoptosis and migration of RCC cells

**Figure 3:**
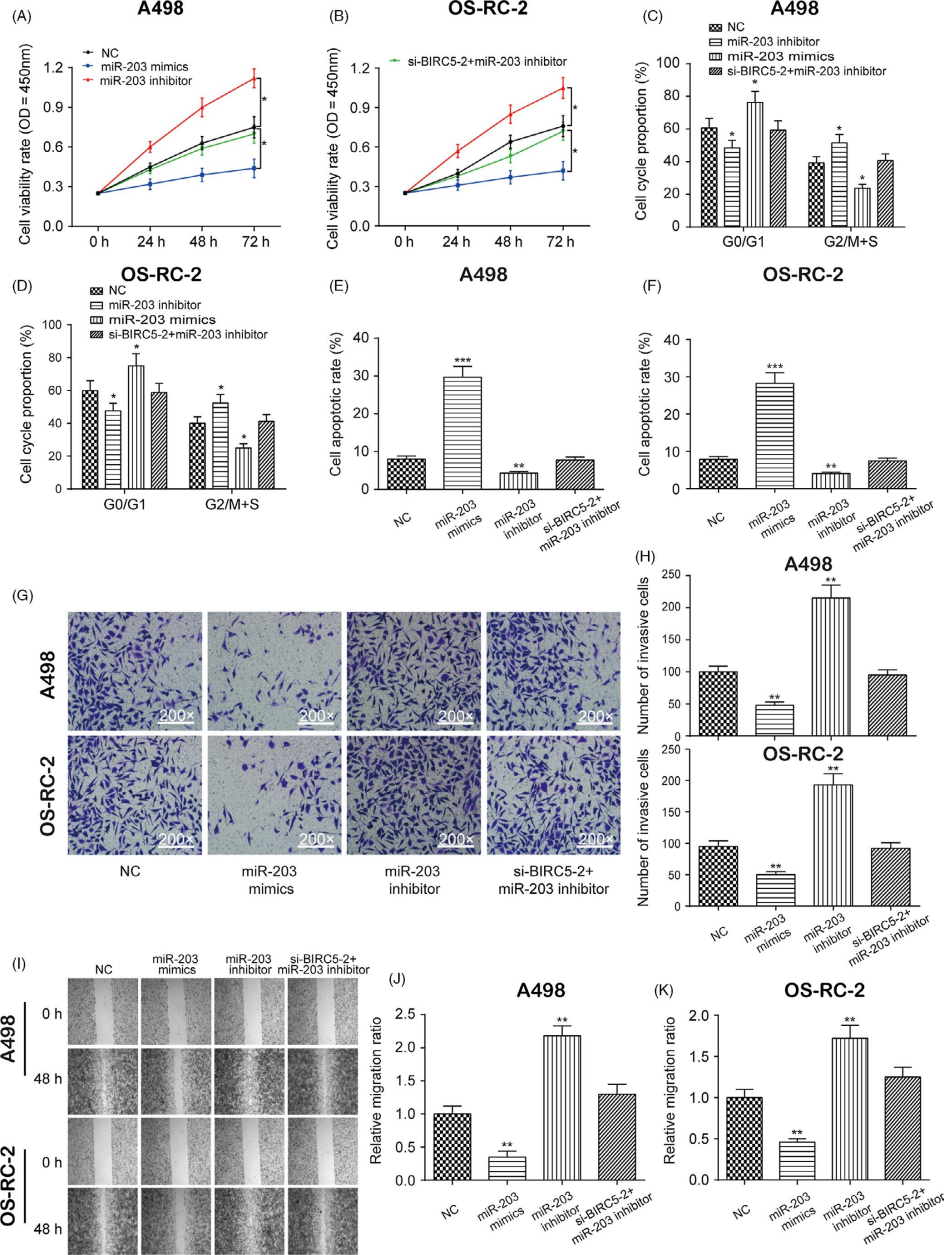
Effects of miR‐203 on cell proliferation, cell cycle, apoptosis, invasion and migration in A498 and OS‐RC‐2 cells. A and B, The effects of miR‐203 on the proliferation of A498 cells and OS‐RC‐2 cells were examined by CCK‐8 assay. C‐F, The effects of miR‐203 on the cell cycle (C and D) and apoptosis (E and F) in A498 cells and OS‐RC‐2 cells were measured by flow cytometry. G‐K, The effects of miR‐203 on the invasion (G and H) and migration (I‐K) of A498 cells and OS‐RC‐2 cells were examined by transwell assay and wound healing assay, respectively. Data were expressed as mean ± standard deviation of three independent experiments. *P < 0.05. **P < 0.01. ***P < 0.001

The CCK‐8 assay indicated that the overexpression of miR‐203 significantly decreased cell viability, which was instead increased with downregulation of miR‐203 in both A498 and OS‐RC‐2 cells (Figure 3A,B, *P < 0.05). In addition, flow cytometry showed that the overexpression of miR‐203 greatly increased the percentage of RCC cells in G0/G1 phase, while the downregulation of miR‐203 decreased the percentage of RCC cells in G0/G1 phase in both A498 and OS‐RC‐2 cells (Figures 3C,D and S4A,B, *P < 0.05). Furthermore, the overexpression of miR‐203 significantly increased cell apoptotic rate, which was instead decreased with the suppression of miR‐203 in both A498 and OS‐RC‐2 cells (Figures 3E,F and S4C,D, **P < 0.01, ***P < 0.001). The results of the transwell (Figure 3G,H, **P < 0.01) and wound healing (Figure 3I‐K, **P < 0.01) assays showed that the suppression of miR‐203 significantly increased cell invasion and migration, which was otherwise decreased with upregulation of miR‐203 in both A498 and OS‐RC‐2 cells. However, the suppression of miR‐203 greatly affected cell proliferation, cell cycle, apoptosis, invasion and migration, which was returned to its normal expression level after co‐transfection with si‐*BIRC5*‐2. In short, miR‐203 affected cell proliferation, cell cycle, apoptosis, invasion and migration by targeting *BIRC5* in RCC.

### 3.4 Targeted relationship between miR‐203 and MALAT1 was validated

**Figure 4:**
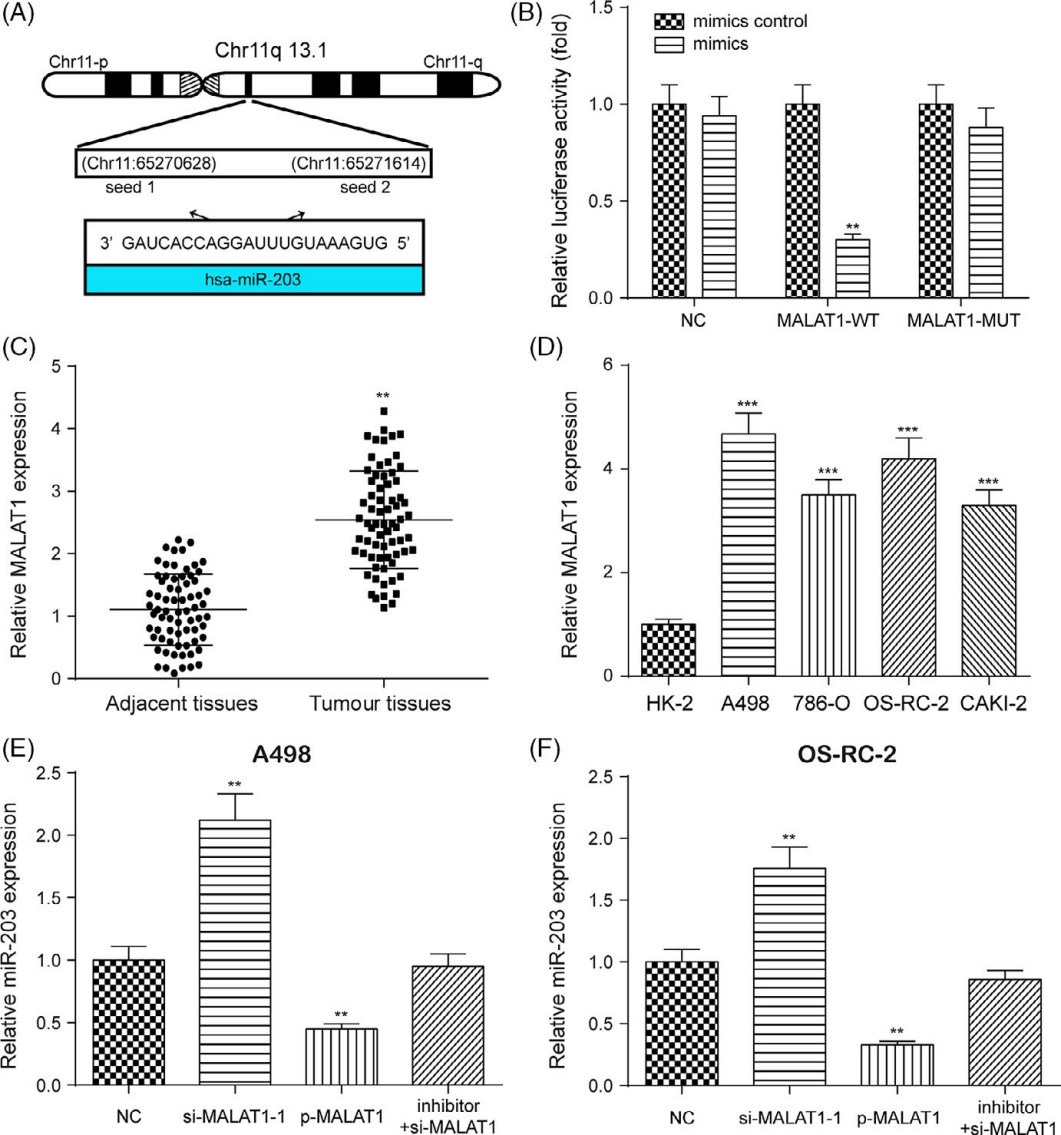
The targeted relationship between miR‐203 and MALAT1 was validated. A, miR‐203 was predicted to target MALAT1 by starBase. B, Dual‐luciferase reporter gene assay validated the targeted relationship between miR‐203 and MALAT1. C and D, The expression of MALAT1 in RCC tissues and cell lines was examined by qRT‐PCR. E and F, The overexpression of MALAT1 greatly downregulated the expression of miR‐203, while the suppression of MALAT1 greatly upregulated the expression of miR‐203 in both A498 and OS‐RC‐2 cells. Data were expressed as mean ± standard deviation of three independent experiments. **P < 0.01. ***P < 0.001

**Figure 5:**
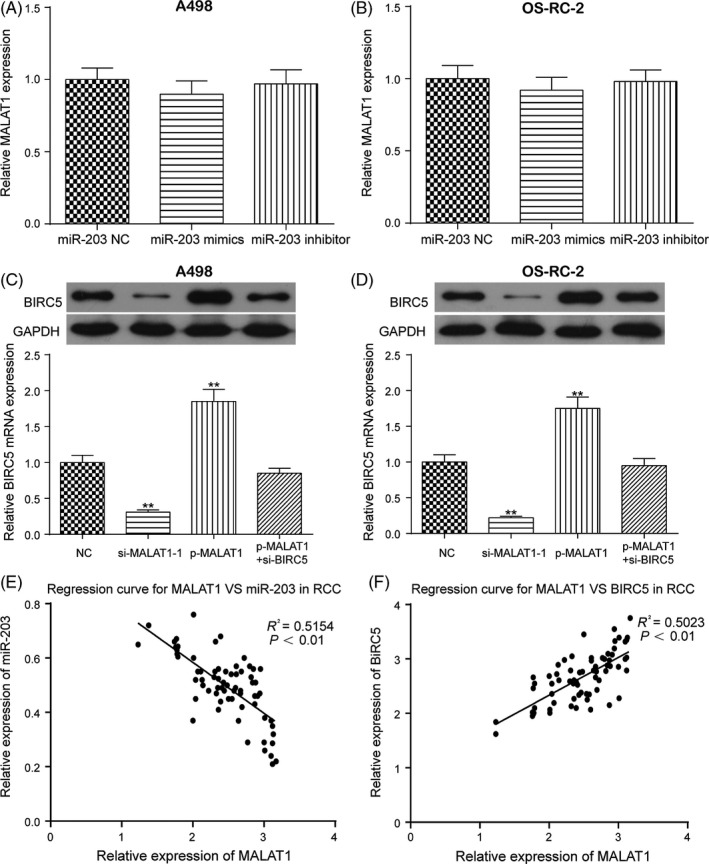
miR‐203 did not affect the expression of MALAT1, and the overexpression of MALAT1 promoted the expression of *BIRC5* in both A498 and OS‐RC‐2 cells. A and B, miR‐203 did not affect the expression of MALAT1 in both A498 and OS‐RC‐2 cells. C and D, The overexpression of MALAT1 promoted the expression of *BIRC5* in both A498 and OS‐RC‐2 cells. E and F, Regression analysis showed that the expression of MALAT1 was negatively correlated with the expression of miR‐203 and positively correlated with the expression of *BIRC5*. Data were expressed as mean ± standard deviation of three independent experiments. **P < 0.01

Two potential binding sites between MALAT1 and miR‐203 were identified based on starBase as shown in Figure 4A. Dual‐luciferase reporter gene assay indicated that the luciferase activity of the group co‐transfected with miR‐203 mimics and MALAT1‐wt was lower than that of the group co‐transfected with miR‐203 NC and MALAT1‐wt (Figure 4B, **P < 0.01), while MALAT1‐mut did not affect luciferase activity, suggesting a targeted relationship between miR‐203 and MALAT1 in RCC. The expression of MALAT1 was higher in RCC tissues and cell lines than in adjacent normal tissues and a normal renal cortex proximal tubule epithelial cell line (Figure 4C,D, **P < 0.01). P‐MALAT1 greatly increased the expression of MALAT1, which was instead decreased with si‐MALAT1‐1 and si‐MALAT1‐2 (Figure S5A, **P < 0.01, ***P < 0.001). Given that the transfection efficiency was higher in the si‐MALAT1‐1 group, si‐MALAT1‐1 was used for further experiments. qRT‐PCR showed that the overexpression of MALAT1 greatly downregulated the expression of miR‐203, which was instead increased with the suppression of MALAT1 in both A498 and OS‐RC‐2 cells (Figure 4E,F, **P < 0.01). The upregulated miR‐203 expression by the MALAT1 inhibition was rescued to its normal expression level after co‐transfection with miR‐203 inhibitor. However, the overexpression or suppression of miR‐203 almost did not affect the expression of MALAT1 in both A498 and OS‐RC‐2 cells (Figure 5A,B). Besides, the upregulation of MALAT1 increased the expression of *BIRC5*, while the downregulation of MALAT1 decreased the expression of *BIRC5* in both A498 and OS‐RC‐2 cells (Figure 5C,D, **P < 0.01). Furthermore, regression analysis revealed that the expression of MALAT1 was negatively correlated with the expression of miR‐203, while the expression of MALAT1 was positively correlated with the expression of *BIRC5* as shown in Figure 5E,F. In brief, the targeted relationship between miR‐203 and MALAT1 was validated. The overexpression of MALAT1 increased the expression of *BIRC5* yet decreased the expression of miR‐203 in RCC.

### 3.5 Effects of MALAT1 on the proliferation, cell cycle, apoptosis and migration of RCC cells

**Figure 6:**
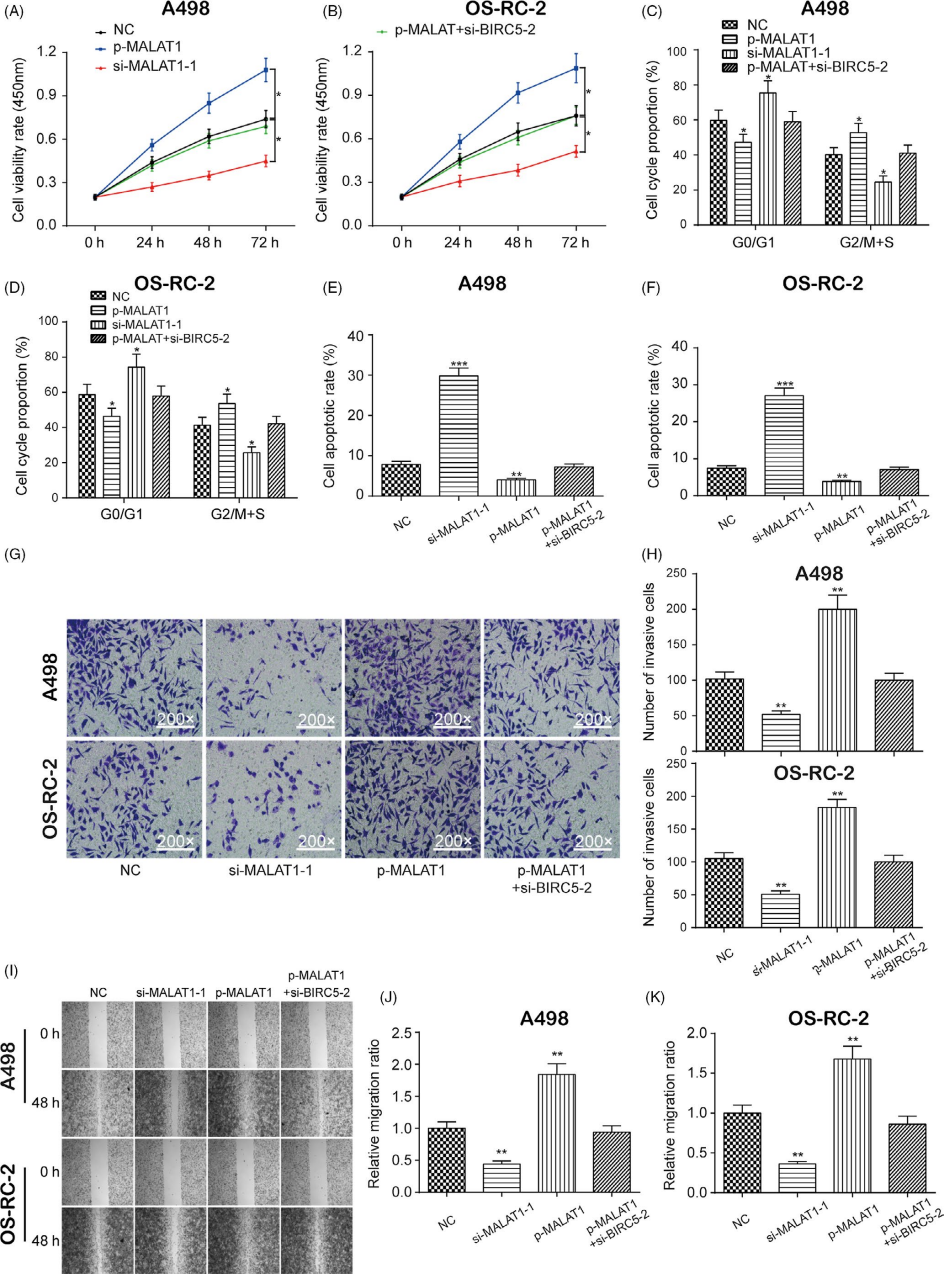
Effects of MALAT1 on cell proliferation, cell cycle, apoptosis, invasion and migration of A498 and OS‐RC‐2 cells. A and B, The effects of MALAT1 on the proliferation of A498 cells and OS‐RC‐2 cells were examined by CCK‐8 assay. C‐F, The effects of miR‐203 on the cell cycle (C and D) and apoptosis (E and F) in A498 cells and OS‐RC‐2 cells were measured by flow cytometry. G‐K, The effects of miR‐203 on the invasion and migration of A498 cells and OS‐RC‐2 cells were examined by transwell assay (G and H) and wound healing assay (I‐K), respectively. Data were expressed as mean ± standard deviation of three independent experiments. *P < 0.05. **P < 0.01. ***P < 0.001

The CCK‐8 assay indicated that the upregulation of MALAT1 significantly increased cell viability, which was otherwise decreased with downregulation of MALAT1 in both A498 and OS‐RC‐2 cells (Figure 6A,B, *P < 0.05). Furthermore, flow cytometry showed that the overexpression of MALAT1 greatly decreased the percentage of RCC cells in G0/G1 phase, while the downregulation of MALAT1 increased the percentage of RCC cells in G0/G1 phase in both A498 and OS‐RC‐2 cells (Figures 6C,D and S6A,B, *P < 0.05). In addition, the overexpression of MALAT1 significantly decreased the cell apoptotic rate, which was instead increased with the inhibition of MALAT1 in both A498 and OS‐RC‐2 cells (Figures 6E,F and S6C,D, **P < 0.01, ***P < 0.001). The transwell (Figure 6G,H, **P < 0.01) and wound healing (Figure 6I‐K, **P < 0.01) assays showed that the overexpression of MALAT1 significantly increased cell invasion and migration, which was otherwise decreased with the inhibition of MALAT1 in both A498 and OS‐RC‐2 cells. Similarity, the upregulation of MALAT1 greatly promoted cell proliferation, cell cycle, apoptosis, invasion and migration, which was recovered to its normal expression level after co‐transfection with si‐*BIRC5*‐2. To conclude, MALAT1 affected cell proliferation, cell cycle, apoptosis, invasion and migration by decreasing the expression of miR‐203 and promoting the expression of *BIRC5* in RCC.

### 3.6 MALAT1 promoted RCC tumorigenesis in vivo

**Figure 7:**
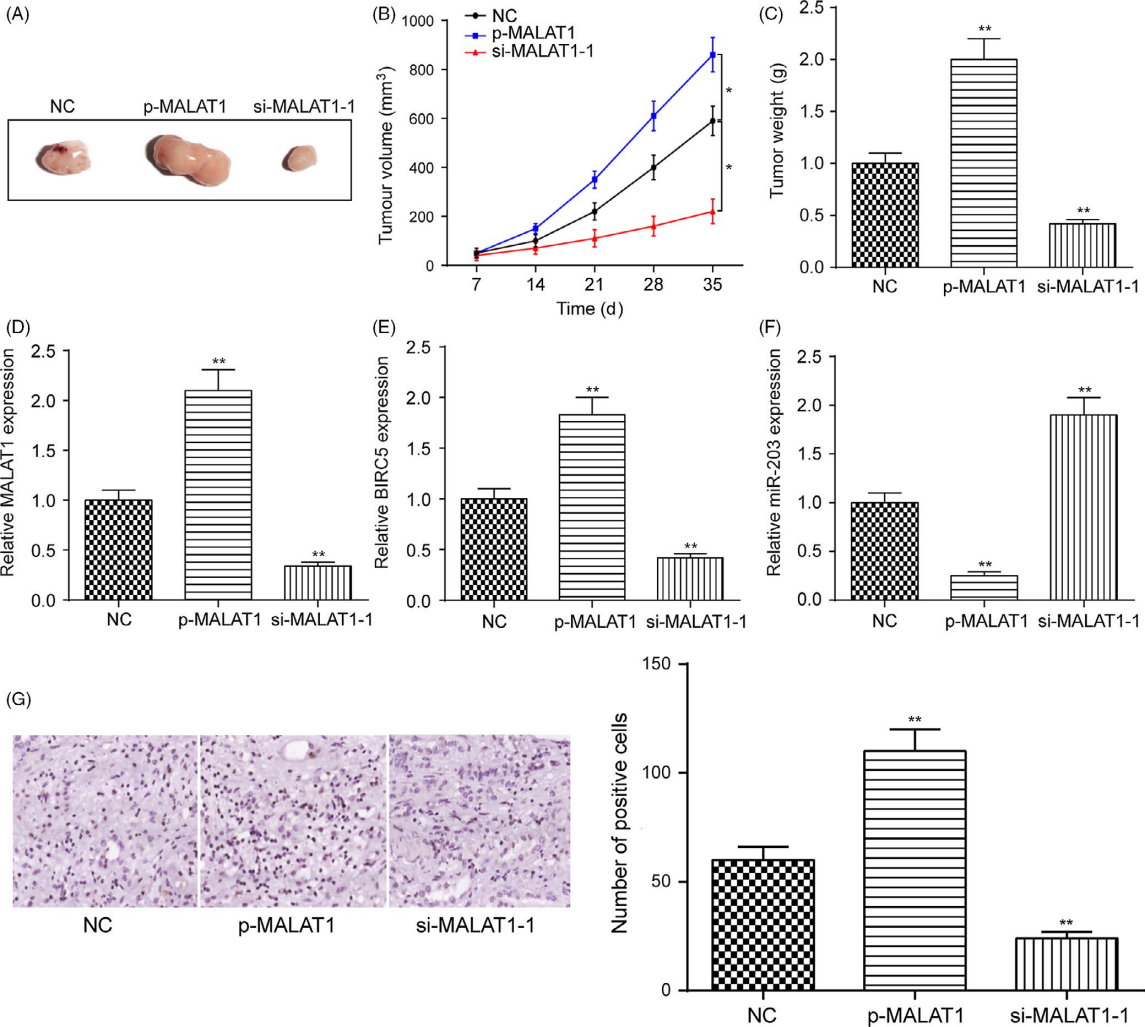
MALAT1 promotes RCC tumorigenesis in vivo. A, Morphology of tumour xenograft. B, The change in tumour volume was determined every 7 d during the tumour growth. C, Tumour weight of nude mice was significantly increased by the transfection with p‐MALAT1 and reduced by the transfection with si‐MALAT1. D‐F, The expression of MALAT1, *BIRC5* and miR‐203 in each group tumour tissues was determined by qRT‐PCR. G, the expression of Ki67 in each group tumour tissues was determined by IHC. Data were expressed as mean ± standard deviation of three independent experiments. *P < 0.05. **P < 0.01

As shown in Figure 7A‐C, the overexpression of MALAT1 promoted tumour growth with A498 cells, while the downregulation of MALAT1 suppressed tumour growth in vivo as evidenced by the tumour volume and weight of nude mice (*P < 0.05, **P < 0.01). Besides, p‐MALAT1 greatly upregulated the expression of MALAT1, which was downregulated with si‐MALAT1‐1 in tumour tissues (Figure 7D, **P < 0.01). Furthermore, the overexpression of MALAT1 greatly increased the expression of *BIRC5* yet downregulated the expression of miR‐203 in tumour tissues, indicating that MALAT1 sponged miR‐203 for the upregulation of *BIRC5* (Figure 7E,F, **P < 0.01). Finally, IHC revealed that the overexpression of MALAT1 greatly upregulated the expression of Ki67, which was downregulated with silencing of MALAT1. In brief, MALAT1 promoted RCC tumorigenesis in vivo*.*

## 4 DISCUSSION

LncRNAs are important regulators of gene expression that interact with major signalling pathways for cell growth, proliferation, differentiation, apoptosis, migration, and invasion.^29^ In some studies, MALAT1 acted as an miR‐203 sponges to promote inflammation in myocardial ischaemia‐reperfusion injury.^30^ In our study, MALAT1 enhanced cell proliferation and migration yet inhibited cell apoptosis by promoting the expression of *BIRC5* yet suppressing the expression of miR‐203. Taken together, our results suggest that MALAT1 accelerated the development and progression of RCC by decreasing the expression of miR‐203 yet promoting the expression of *BIRC5*.

*BIRC5* levels were higher in RCC tissues than in adjacent normal tissues, and patients expressing high levels of *BIRC5* presented a poor prognosis in our study. Higher expression level of *BIRC5* was detected in four RCC cell lines (A498, 786‐O, OS‐RC‐2, CAKI‐1) than in a normal renal cortex proximal tubule epithelial cell line. *BIRC5* has been shown to inhibit cell apoptosis yet promote cell proliferation in human cancers. The overexpression of *BIRC5* was observed in almost all human malignancies, and the increased expression of *BIRC5* was correlated with poor clinical outcomes, tumour recurrence and drug resistance in cancer patients.^31^ Recently, increasing numbers of studies have showed that *BIRC5* is regulated by cytokines in lymphocytes and plays a vital role in the proliferation and survival of haematopoietic cells.^32^ We verified that *BIRC5* accelerated cell proliferation and the cell cycle, inhibited apoptotic pathways and promoted cell migration. Some articles also showed that *BIRC5* inhibited caspase‐dependent apoptotic pathways and caspase‐independent apoptotic pathways as well as accelerated cell proliferation.^20^

We investigated proposed targeted relationship between miR‐203 and *BIRC5*. Our results showed that the overexpression of miR‐203 inhibited *BIRC5* expression, while the inhibition of an miR‐203 inhibitor accelerated *BIRC5* expression. Wang et al demonstrated that miR‐203 suppressed the proliferation and migration of lung cancer cells and promoted their apoptosis by targeting *SRC*.^30^ Zhang et al suggested that miR‐203 inhibited tumour growth and invasion in oesophageal cancer by inhibiting *Ran*.^6^ These studies are generally consistent with our results. In addition, we verified that miR‐203 inhibited cell proliferation, cell cycle progression and cell migration but promoted apoptotic pathways by targeting *BIRC5*.

Moreover, MALAT1 was shown to be upregulated in RCC. MALAT1 upregulated *BIRC5* expression to promote cell proliferation, cell cycle progression and migration yet inhibited cell apoptosis in RCC through targeting miR‐203. Besides, MALAT1 promoted OS tumour growth *in vivo*.^33^, ^34^ Furthermore, MALAT1 regulated ovarian cancer cell proliferation, migration and apoptosis through Wnt/β‐catenin signalling pathway.^35^

Our research showed that MALAT1 promoted RCC tumorigenesis in vitro. We validated that the expression level of MALAT1 was higher in RCC tissues and cell lines than in adjacent normal tissues and a normal renal cortex proximal tubule epithelial cell line. RCC patients with high MALAT1 expression had more advanced clinical features and a shorter overall survival time than those with low MALAT1 expression.^36^ MALAT1 promoted cancer cell proliferation and metastasis through activating the ERK/MAPK pathway and interacting with hnRNP during cell cycle regulation.^19^ In the same way, our study explored the mechanism of action between them.

To conclude, we confirmed the direct targeted relationships between MALAT1 and miR‐203, as well as miR‐203 and *BIRC5*. Besides, the downregulation of MALAT1 significantly increased the expression of miR‐203, which was consistent with the results of Chen et al^37^ However, upregulation of MALAT1 greatly decreased the expression of miR‐203. MALAT1 regulated hypoxia‐induced angiogenesis, activated ERK/MAPK signal transduction and eliminated anti‐hypertrophic microRNA‐133.^38^

However, the limitations of this study should be taken into account. It was of great importance to detect relevant factors or signalling pathways that could be employed for further validation of significant roles of MALAT1/miR‐203/*BIRC5* in RCC. In our study, the expression of both survivin and cell proliferation marker Ki67 was detected by IHC as shown in Figures 1B and 7G, respectively, to validate the significant roles of MALAT1/miR‐203/ *BIRC5* in the development and progression of renal cell carcinoma. However, other relevant markers and pathways, such as caspase 3/9, cyclins or EMT, will be measured and validated in further studies. In addition, uncovering more targets of miR‐203 in RCC could facilitate therapeutic intervention in the future. Furthermore, MALAT1 was reported to be closely related to the development and progression of a variety of human cancers. MALAT1 was reported to be overexpressed in several kinds of human cancers, such as osteosarcoma,^34^ lung cancer^39^ and colorectal cancer.^40^ The detection of MALAT1 has not yet been utilized for the diagnosis and treatment of RCC. However, upgraded and integrated therapies could be developed based on the detection of MALAT1. More miRNAs specifically targeting MALAT1 in RCC needed to be uncovered for the development of integrated therapies. In addition, more specific mRNAs regulated by MALAT1 in RCC could be selected as potential therapeutic targets.

In conclusion, MALAT1 accelerates the development and progression of renal cell carcinoma by decreasing the expression of miR‐203 and promoting the expression of *BIRC5*.

## CONFLICT OF INTEREST

The authors declare that they have no conflict of interest.

## AUTHOR CONTRIBUTIONS

HMZ and WL designed the study; WYG and YY collected the data; HMZ and XDY analysed and interpreted the data; and JHZ involved in critical reviewing of the manuscript.

## ETHICS APPROVAL

This study was approved by Shanghai General Hospital, The First People's Hospital Affiliated to Shanghai Jiaotong University.

## Data Availability

The data that support the findings of this study are available from the corresponding author upon reasonable request.
