# Supplementary figures and images for: MALAT1 accelerates the development and progression of renal cell carcinoma by decreasing the expression of miR‐203 and promoting the expression of BIRC5
Source: Cell Prolif. 2019 Jun 27;52(5):e12640. doi: 10.1111/cpr.12640 (PMC6797509; doi:10.1111/cpr.12640)

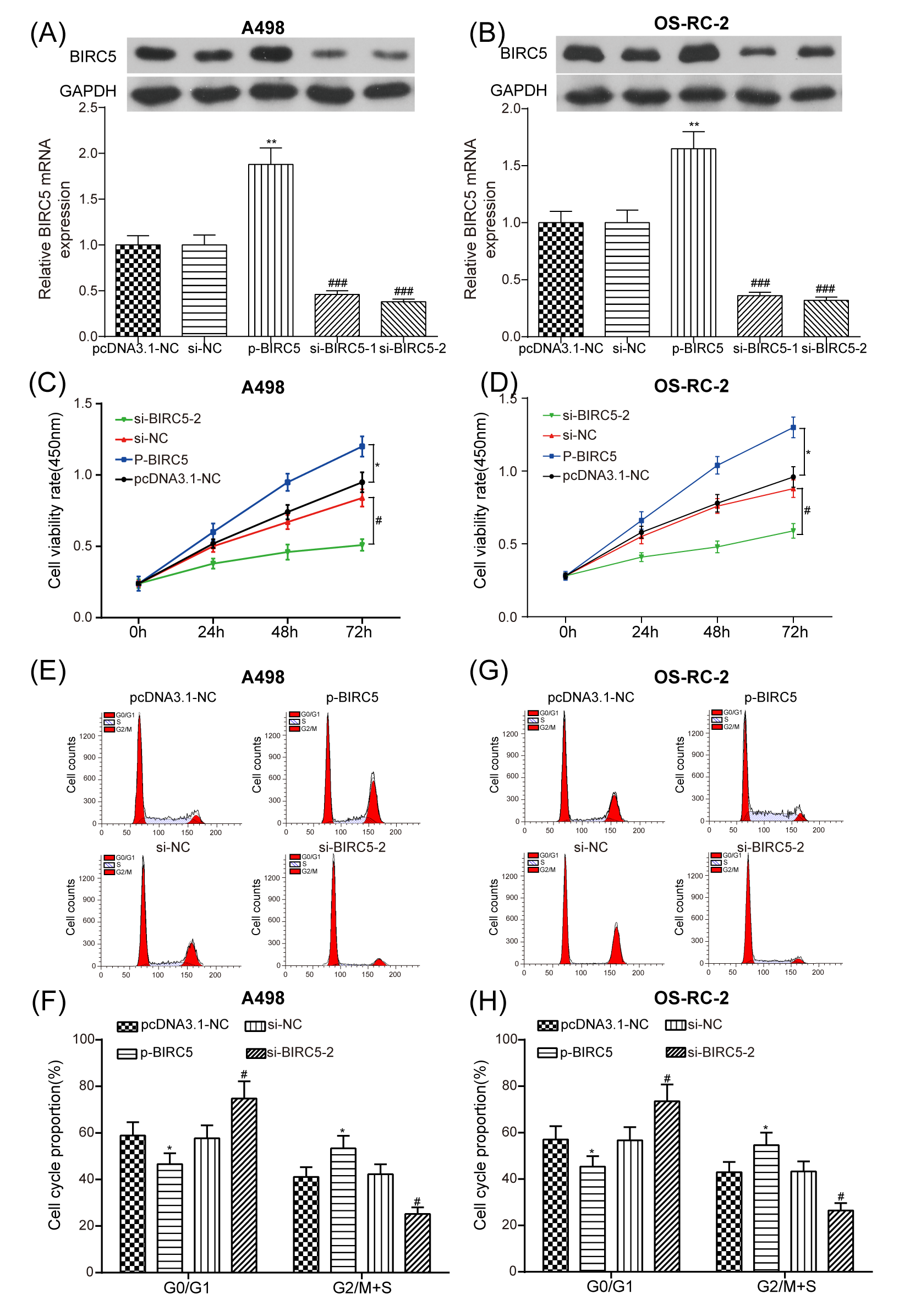

Supplement: Supplementary file 1 [file CPR-52-e12640-s001.tif]

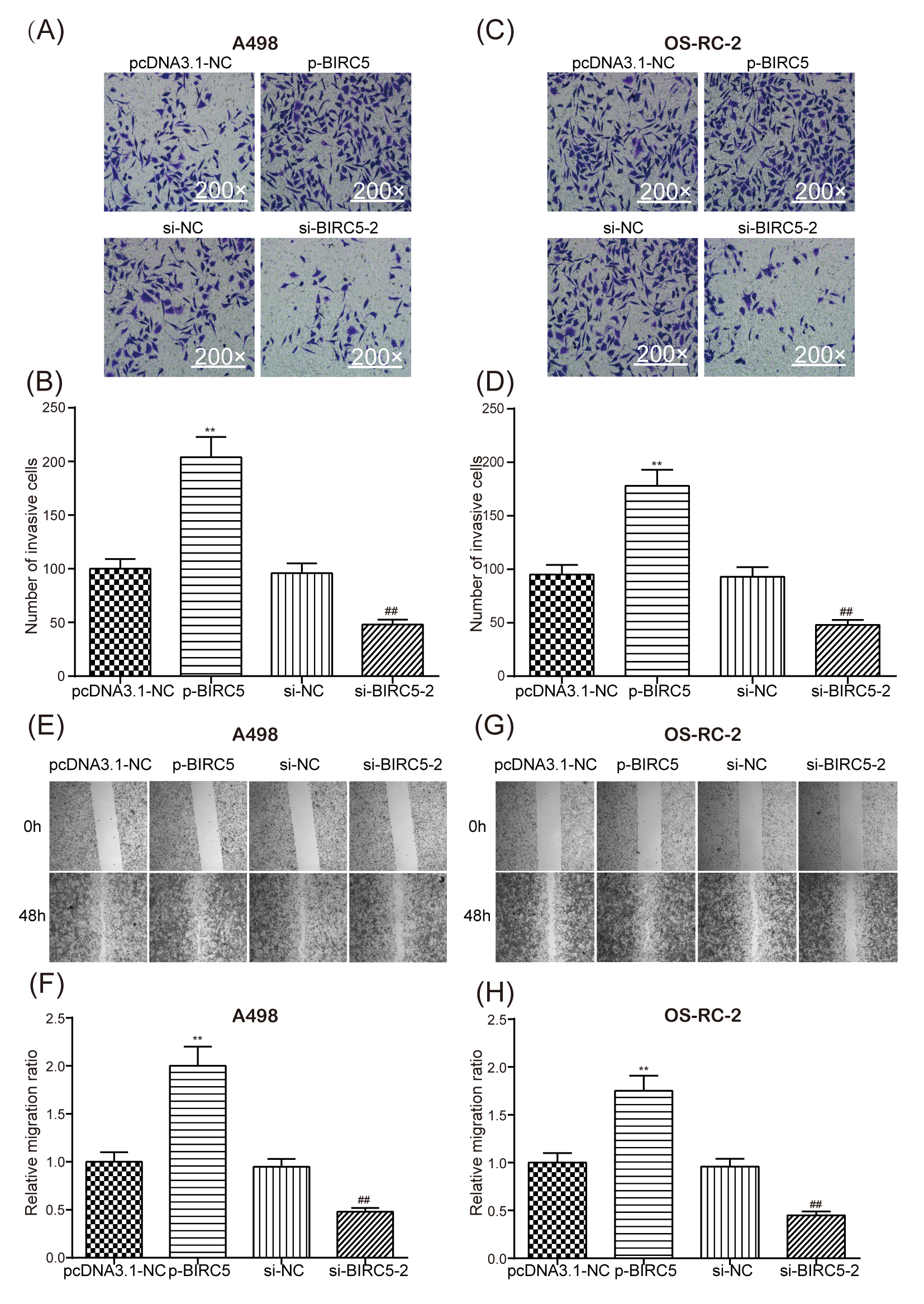

Supplement: Supplementary file 2 [file CPR-52-e12640-s002.tif]

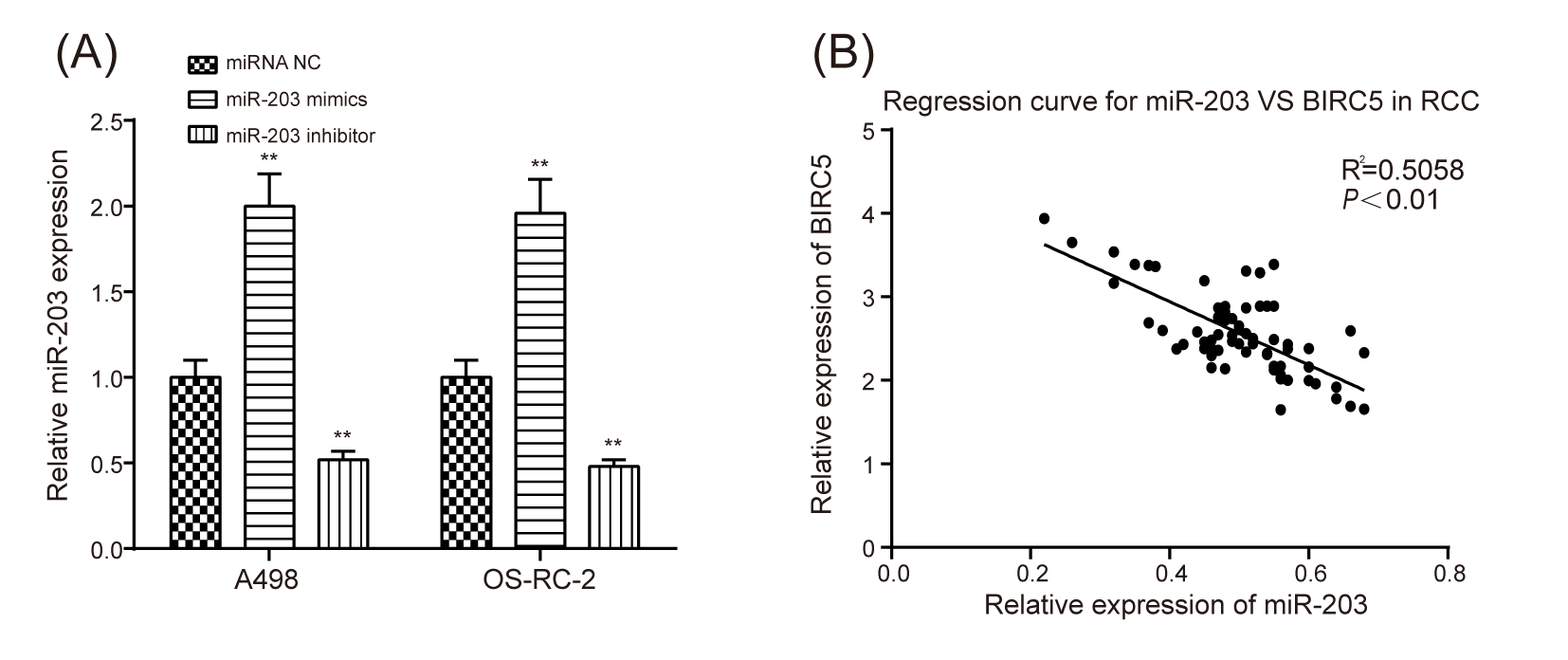

Supplement: Supplementary file 3 [file CPR-52-e12640-s003.tif]

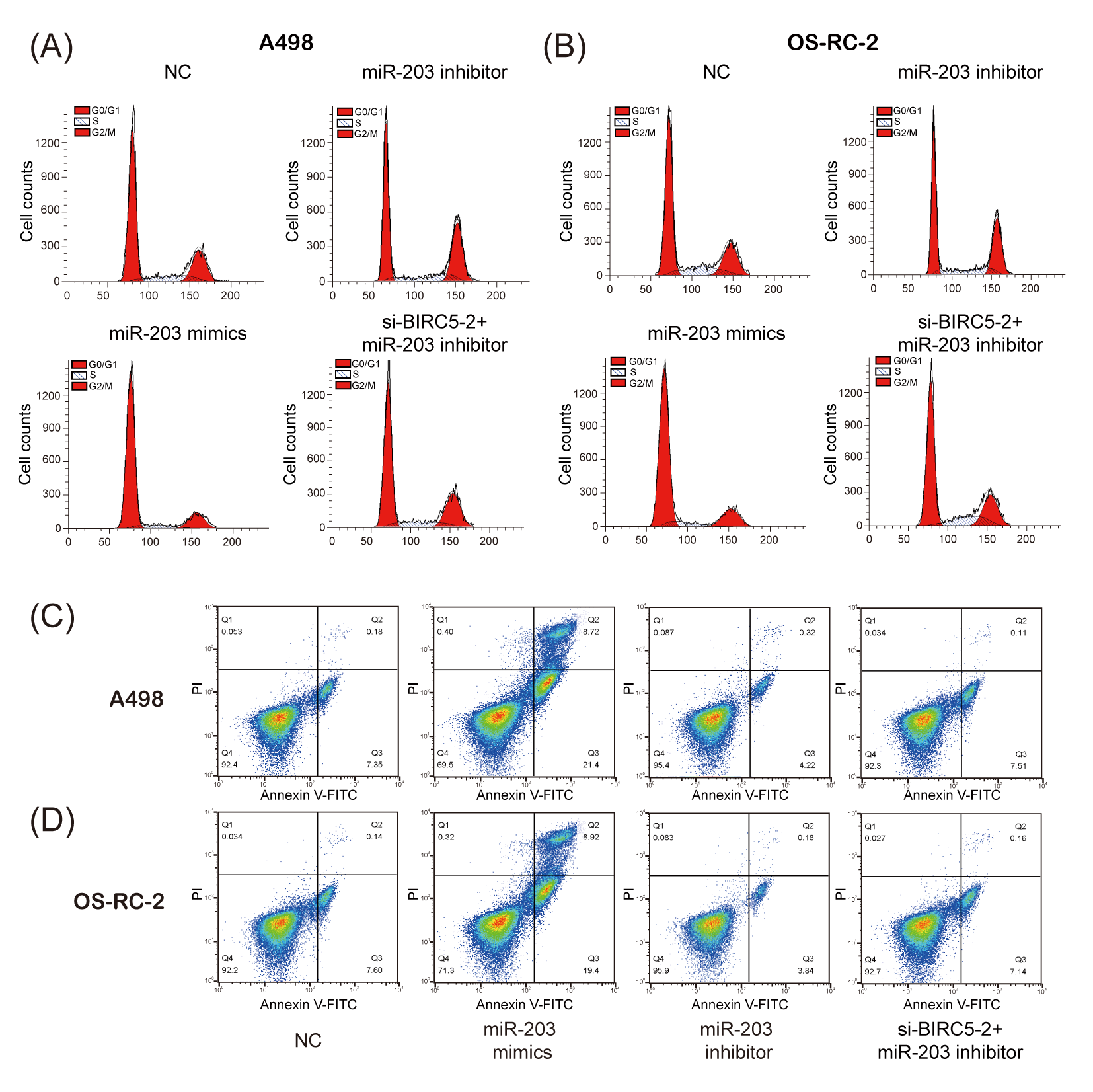

Supplement: Supplementary file 4 [file CPR-52-e12640-s004.tif]

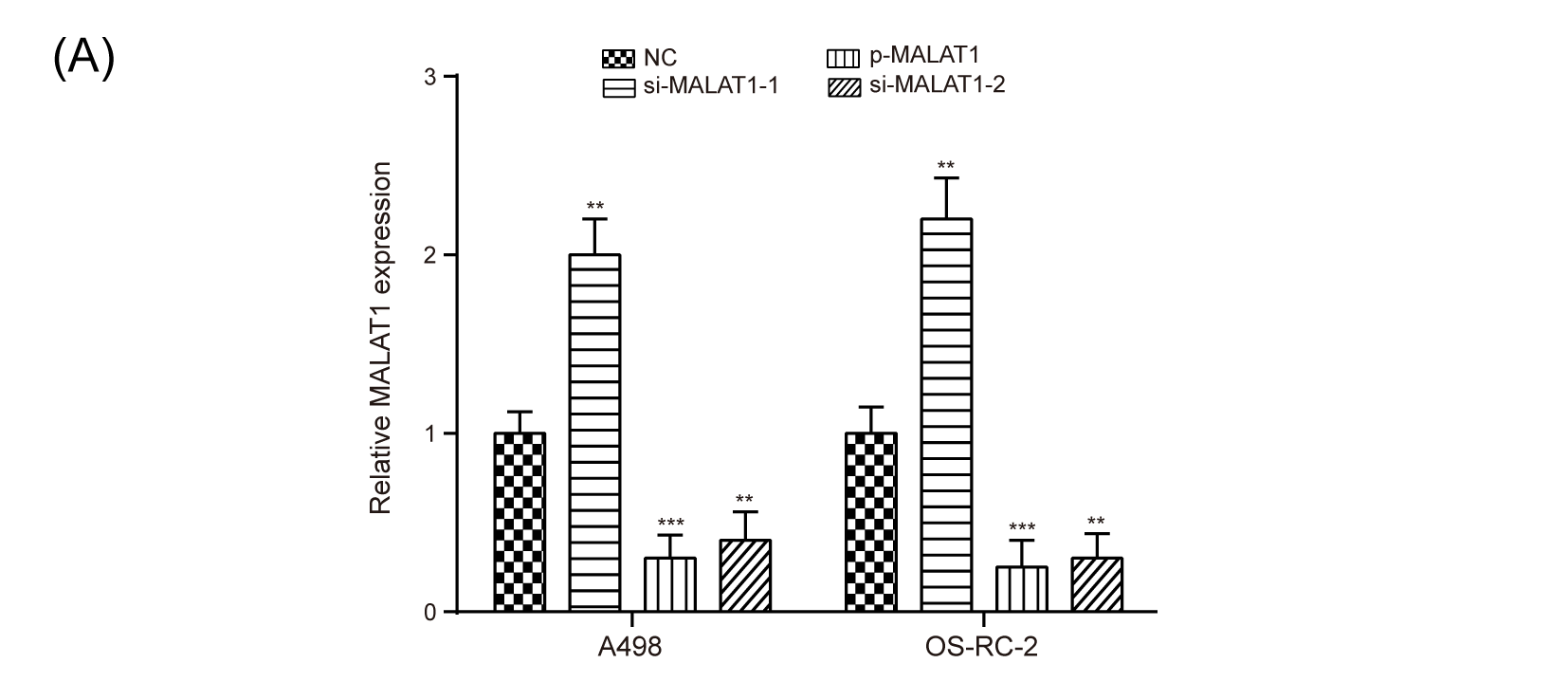

Supplement: Supplementary file 5 [file CPR-52-e12640-s005.tif]

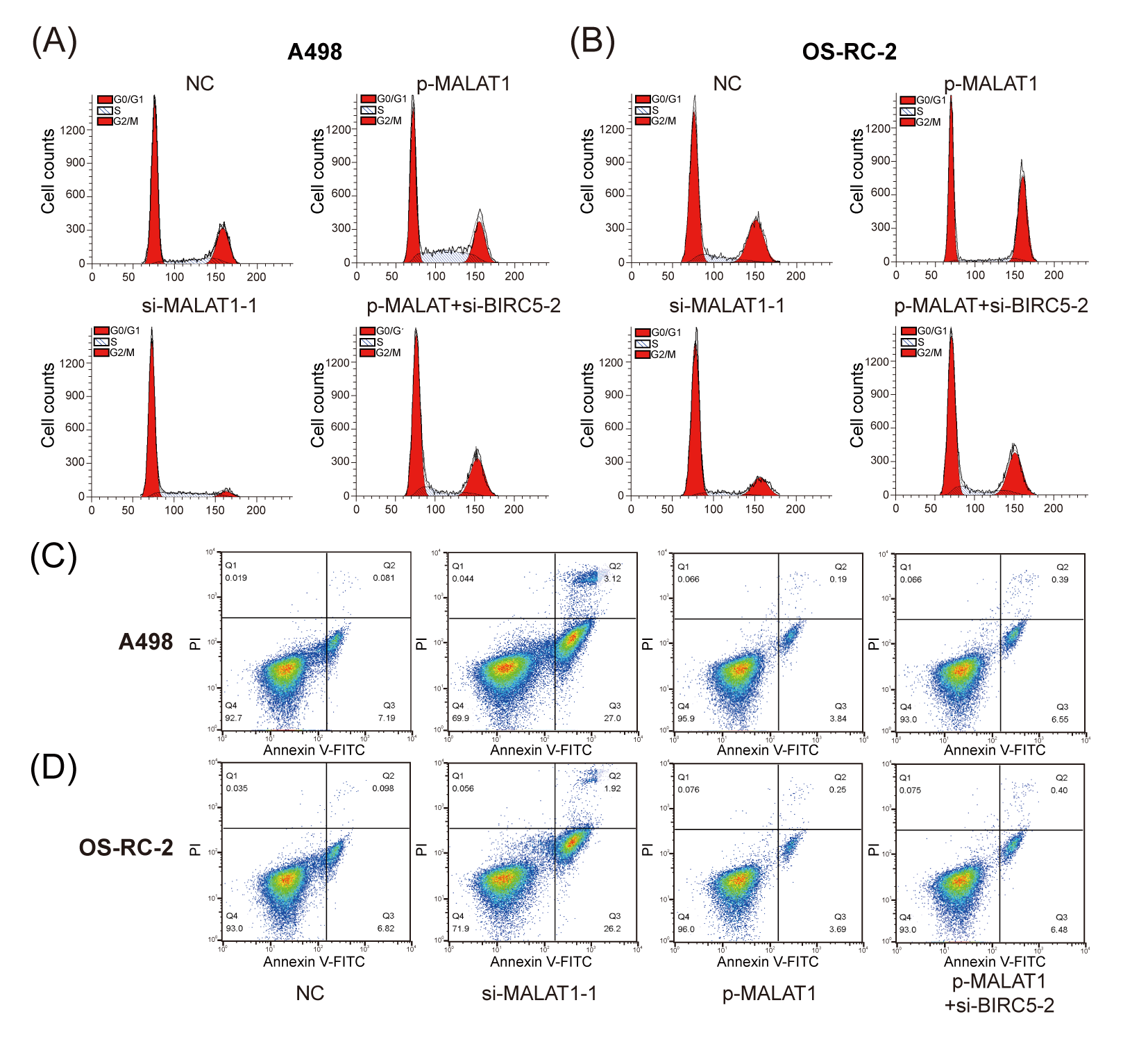

Supplement: Supplementary file 6 [file CPR-52-e12640-s006.tif]
