# Supplementary material for: MALAT1 accelerates the development and progression of renal cell carcinoma by decreasing the expression of miR‐203 and promoting the expression of BIRC5
Source: Cell Prolif. 2019 Jun 27;52(5):e12640. doi: 10.1111/cpr.12640 (PMC6797509; doi:10.1111/cpr.12640)
Supplement: Supplementary file 7 [file CPR-52-e12640-s007.docx]

Table S1 Primers for qRT-PCR

| Name | Forward primers 5′-3′ | Reverse primers 5′-3′ | |
| --- | --- | --- | --- |
| MALAT1 | GGGTGTTTACGTAGACCAGAACC | | CTTCCAAAAGCCTTCTGCCTTAG |
| *BIRC5* | AGGACCACCGCATCTCTACAT | | AAGTCTGGCTCGTTCTCAGTG |
| miR-203 | GATCACCAGGATTTGTAA | | GTATCCAGTGCGAATACCTC |
| U6 | ATTGGAACGATACAGAGAAGATT | | GGAACGCTTCACGAATTTG |
| GAPDH | AGACAGCCGCATCTTCTTGT | | TGATGGCAACAATGTCCACT |
