# Supplementary material for: MALAT1 accelerates the development and progression of renal cell carcinoma by decreasing the expression of miR‐203 and promoting the expression of BIRC5
Source: Cell Prolif. 2019 Jun 27;52(5):e12640. doi: 10.1111/cpr.12640 (PMC6797509; doi:10.1111/cpr.12640)
Supplement: Supplementary file 8 [file CPR-52-e12640-s008.docx]

Table S2 The sequences used for transfection

| Name | Sequence |
| --- | --- |
| Si-BIRC5-1 | CCCAGUGUUUCUUCUGCUU |
| Si-BIRC5-2 | GCGCUUUCCUUUCUGUCAA |
| Mimics | AGUGGUUCUUAACAGUUCAACAGUU |
| Inhibitor | UCACCAAGAAUUGUCAAGUUGUCAA |
